# Activation of ERK1/2 by Store-Operated Calcium Entry in Rat Parotid Acinar Cells

**DOI:** 10.1371/journal.pone.0072881

**Published:** 2013-08-29

**Authors:** Stephen P. Soltoff, William A. Lannon

**Affiliations:** Beth Israel Deaconess Medical Center, Department of Medicine, Division of Signal Transduction, Harvard Medical School, Boston, Massachussetts, United States of America; University of Tennessee Health Science Center, United States of America

## Abstract

The regulation of intracellular Ca^2+^ concentration ([Ca^2+^]_i_) plays a critical role in a variety of cellular processes, including transcription, protein activation, vesicle trafficking, and ion movement across epithelial cells. In many cells, the activation of phospholipase C-coupled receptors hydrolyzes membrane phosphoinositides and produces the depletion of endoplasmic reticulum Ca^2+^ stores, followed by the sustained elevation of [Ca^2+^]_i_ from Ca^2+^ entry across the plasma membrane via store-operated Ca^2+^ entry (SOCE). Ca^2+^ entry is also increased in a store-independent manner by arachidonate-regulated Ca^2+^ (ARC) channels. Using rat parotid salivary gland cells, we examined multiple pathways of Ca^2+^ entry/elevation to determine if they activated cell signaling proteins and whether this occurred in a pathway-dependent manner. We observed that SOCE activates extracellular signal-related kinases 1 and 2 (ERK1/2) to ∼3-times basal levels via a receptor-independent mechanism when SOCE was initiated by depleting Ca^2+^ stores using the endoplasmic reticulum Ca^2+^-ATPase inhibitor thapsigargin (TG). TG-initiated ERK1/2 phosphorylation increased as rapidly as that initiated by the muscarinic receptor agonist carbachol, which promoted an increase to ∼5-times basal levels. Notably, ERK1/2 phosphorylation was not increased by the global elevation of [Ca^2+^]_i_ by Ca^2+^ ionophore or by Ca^2+^ entry via ARC channels in native cells, although ERK1/2 phosphorylation was increased by Ca^2+^ ionophore in Par-C10 and HSY salivary cell lines. Agents and conditions that blocked SOCE in native cells, including 2-aminoethyldiphenyl borate (2-APB), SKF96363, and removal of extracellular Ca^2+^, also reduced TG- and carbachol-stimulated ERK1/2 phosphorylation. TG-promoted ERK1/2 phosphorylation was blocked when SRC and Protein Kinases C (PKC) were inhibited, and it was blocked in cells pretreated with β-adrenergic agonist isoproterenol. These observations demonstrate that ERK1/2 is activated by a selective mechanism of Ca^2+^ entry (SOCE) in these cells, and suggest that ERK1/2 may contribute to events downstream of SOCE.

## Introduction

Receptor-mediated increases in [Ca^2+^]_i_ promote a variety of physiological events in many cells, including the stimulation of fluid and electrolyte secretion in salivary gland epithelial cells [1], [2], [3]. Cells utilize multiple mechanisms of Ca^2+^ entry. In various non-excitable cells, the increases in [Ca^2+^]_i_ involve extracellular Ca^2+^ entry into the cell via SOCE, which is initiated by the release of Ca^2+^ stores from the endoplasmic reticulum via inositol 1,4,5-trisphosphate receptors (IP_3_R)/Ca^2+^ channels when the activation of G-protein-coupled receptors produces IP_3_ and diacylglycerol from phosphatidylinositol-4,5-bisphosphate (PIP_2_) hydrolysis by phospholipase C [4]. In contrast, at low concentrations of receptor agonists, Ca^2+^ signals may be initiated by ARC channels, which produce oscillatory increases in [Ca^2+^]_i_ rather than the sustained increases in [Ca^2+^]_i_ that are produced by SOCE. ARC channels rely on the generation of arachidonic acid and are store-independent, since their activation does not depend on the loss of Ca^2+^ from the endoplasmic reticulum [5], [6]. In addition to Ca^2+^-sensitive ion movements that occur in response to Ca^2+^ entry via the SOCE pathway in salivary gland cells, the entry of extracellular Ca^2+^ via the P2X_7_ receptor/ion channel also activates Ca^2+^-sensitive ion channels and initiates fluid secretion and saliva formation [7], [8], [9]. SOCE can be activated in a receptor-independent manner using agents such as TG that block the Ca^2+^-ATPase on the endoplasmic reticulum membrane, which depletes endoplasmic reticulum Ca^2+^ stores and thereby promotes Ca^2+^ entry.

The stimulation of fluid secretion in salivary gland cells involves Ca^2+^-sensitive K^+^ and Cl^−^ channels which are opened downstream of receptor-mediated [Ca^2+^]_i_ elevation via SOCE, and these channels also are opened when [Ca^2+^]_i_ is increased more directly via Ca^2+^ ionophores [2], [3], [10], [11], [12]. Receptor-dependent and receptor-independent increases in [Ca^2+^]_i_ can also initiate signaling cascades, in some cases by transactivating receptors or by increasing the phosphorylation of signaling proteins. The signaling and physiological events downstream of an increase in [Ca^2+^]_i_ may be activated uniquely by a specific mechanism of Ca^2+^ entry and elevation. For example, Ca^2+^-sensitive Adenylyl Cyclase 8 (AC8) is stimulated by the entry of extracellular Ca^2+^ into cells via SOCE but not by ARC channels, Ca^2+^ release from intracellular stores, or global increases in [Ca^2+^]_i_ [13], [14]. In contrast, some Ca^2+^-dependent changes in the phosphorylation of cell signaling proteins can be promoted in similar fashion by both receptor ligands and Ca^2+^ ionophores [15], [16].

The depletion of Ca^2+^ from the endoplasmic reticulum has long been known to promote Ca^2+^ influx across the plasma membrane via SOCE (see [4] for review). SOCE contributes to the regulation of oxidative stress by mitochondria [17], and it is critical for mouse embryonic stem cells to maintain their capacity for self-renewal [18]. In addition, SOCE is required for the normal proliferation of various cells [18], [19]. The molecular nature of SOCE varies in different types of cells, and includes combinations of the following proteins: transient receptor potential C (TRPC) and Orai family members, which are Ca^2+^ channels in the plasma membrane, and stromal interaction molecules (STIM), which serve as Ca^2+^ sensors that links the plasma membrane Ca^2+^ channels to the endoplasmic reticulum Ca^2+^ stores [1], [20], [21], [22]. STIM1 and TRPC family members form the I_CRAC_ current (Ca-release-activated current) and STIM1, Orai1, and TRPC proteins form different Ca^2+^ influx pathways that involve different complexes of these proteins [1], [23], [24]. Other binding partners of the Ca^2+^ channel/STIM1 complex have been found, including Golli-BG21, IP_3_ receptors, and other proteins [24], [25], [26]. Additionally, Orai proteins and STIM1 also are components of ARC channels [6], [27].

Rat parotid acinar cells have been used as a model system to study receptor-initiated Ca^2+^ homeostasis, the activation of cell signaling proteins, and the stimulation of epithelial ion transport mechanisms and fluid secretion [2], [3], [10], [28]. In the present study, we hypothesized that Ca^2+^ elevation might initiate signaling effects downstream of specific Ca^2+^ entry pathways into native parotid cells. In particular, having observed in early studies that SOCE produced an increase in ERK1/2 phosphorylation, we sought to examine whether similar effects were produced by the elevation of [Ca^2+^]_i_ by other mechanisms of Ca^2+^ entry/elevation, including ARC channels and Ca^2+^ ionophore. We also examined the dependence of ERK1/2 activation on various signaling proteins. To gain a more complete understanding of the contributions SOCE to ERK1/2 activation, we compared the effects of receptor-independent activation of SOCE to that of muscarinic receptor activation in a variety of conditions.

## Materials and Methods

### Ethics Statement

The investigation conforms to the “Guide for the Care and Use of Laboratory Animals” published by the National Institutes of Health (NIH Publication No. 85-23, Revised 1996). Our protocol was submitted to and approved by the Institutional Animal Care and Use Committee (Beth Israel Deaconess Medical Center).

### Materials

Carbamyl choline (carbachol) (C4382), isoproterenol (I5627), and 2′(3′)-O-(4-Benzoylbenzoyl) adenosine 5′-triphosphate triethylammonium salt (BzATP, B6396) were purchased from Sigma-Aldrich. Thapsigargin (1138), ionomycin (1704), PP2 (1407), GF109203X (0741), Go6976 (2293), and SKF96363 (1147) were purchased from Tocris Bioscience. Arachidonic acid (Enzo Life Sciences, BML-FA003) and prostaglandin E_2_ (PGE_2_) (Cayman Chemical, 14010) were dissolved in cell culture grade dimethyl sulfoxide (D2650) from Sigma-Aldrich. 2-APB (100065) and phorbol 12-myristate 13-acetate (PMA, 524400) were obtained from EMD Millipore. Fura-2 AM was purchased from Invitrogen/Molecular Probes. Polyclonal ERK2 (SC-154) was purchased from Santa Cruz Biotechnology, Inc. Phospho-Thr202/Tyr204-ERK1/2 (9101) and Phospho-(Ser) PKC substrate (2261) antibodies were purchased from Cell Signaling Technology. Anti-rabbit IgG (AP307P) secondary antibody used for Western blotting was obtained from Chemicon. All other chemicals were reagent grade or better.

### Salivary Gland Acinar Cell Preparations and Solutions

Parotid acinar cells were prepared from male Sprague-Dawley rats (Charles River Laboratories, Kingston, NY, 150–200 g) as described previously [11]. Cells were suspended at ∼0.5–1 mg protein/ml in Solution A composed of the following: 116.4 mM NaCl, 5.4 mM KCl, 1 mM NaH_2_PO_4_, 25 mM Na HEPES, 1.8 mM CaCl_2_, 0.8 mM MgCl_2_, 5 mM Na butyrate, 5.6 mM glucose, pH 7.4. In experiments using BzATP, this solution was modified to contain 1 mM CaCl_2_ without MgCl_2_. Aliquots (1.5 ml) of cells were equilibrated for ∼5–10 min prior to treatments with various agents or vehicles.

### Salivary Gland Cell Lines

Par-C10 cells were grown to near confluence in DMEM-F12 (1∶1) medium containing 2.5% fetal bovine serum and supplements as previously described [29]. HSY cells were grown to near confluence in DMEM medium containing 10% fetal bovine serum. Cells were cultured on BD Falcon tissue culture dishes in a humidified atmosphere of 95% air- 5% CO_2_ at 37°C.

### Western Blot Analysis

Native rat salivary gland cells were treated at 37°C with various agents as indicated in the figure legends, and control cells were exposed to vehicle (dimethyl sulfoxide or H_2_O) for similar periods of time. At the end of the treatment period, the suspended native parotid cells were collected by rapid sedimentation. Cells were lysed in ice-cold lysis buffer (137 mM NaCl, 20 mM Tris base, pH 7.5, 1 mM EGTA, 1 mM EDTA, 10% (v/v) glycerol, 1% v/v Igepal) containing protease and phosphatase inhibitors [30]. The lysates were sedimented at 15,000×g for 15 min at 4 ^o^C. For experiments conducted using cell lines, the cells were treated as indicated in a 37°C incubator for the appropriate times. The cells were removed from the incubator, washed 3 times in phosphate-buffered saline solution, lysed, and sedimented at 15,000×g. The cleared supernatants were diluted with 5× Laemmli sample buffer, boiled for 5 min, and stored at −20°C prior to electrophoresis. Samples were separated using SDS-polyacrylamide gel electrophoresis with an 8% separating gel and a 3% stacking gel. Proteins were transferred to nitrocellulose. Immunoblots were probed overnight with various antibodies according to the supplier’s specifications. Proteins were visualized on X-ray film using enhanced chemiluminescence reagents, and proteins were quantified by densitometry using the Image J software program from the National Institutes of Health as described previously [30]. For each sample, the level of phosphorylated ERK1/2 was normalized to total ERK2 protein or β-actin, and changes in ERK1/2 phosphorylation for various conditions were compared with the phosphorylation status under basal (vehicle) conditions in the absence of stimuli or inhibitors.

### Intracellular Calcium

Alterations in [Ca^2+^]_i_ were analyzed at room temperature in dispersed parotid cells in suspension by measuring changes in the fluorescence of the Ca^2+^-indicator dye Fura-2 as in previous studies [31]. Fura-2-loaded cells were suspended in Solution A and maintained on ice. Where indicated, cells were suspended in the absence of extracellular Ca^2+^ (Ca^2+^-free Solution A containing 10–20 µM EGTA).

### Data Analysis

Values were calculated as the mean±S.E. of *n* number of independent experiments (each *n* from a different cell preparation). ANOVA with Bonferroni correction was used to analyze the significance of differences in data sets of more than two groups. Differences between control/basal and experimental samples were evaluated using Student’s *t* test in two-group data sets. All experiments were performed at least 3 different times. Representative Western blots from one experiment are shown in each figure. Within each experiment to be analyzed using Western blotting techniques, multiple (duplicate or triplicate) cell samples were often collected for each condition, subjected to SDS-PAGE, and the average of the values obtained within each individual experiment were treated as n = 1.

## Results

### SOCE, but not ARC- and Ionophore-mediated Ca^2+^ Entry, Activates ERK1/2 in Rat Parotid Acinar Cells

**Figure 1:**
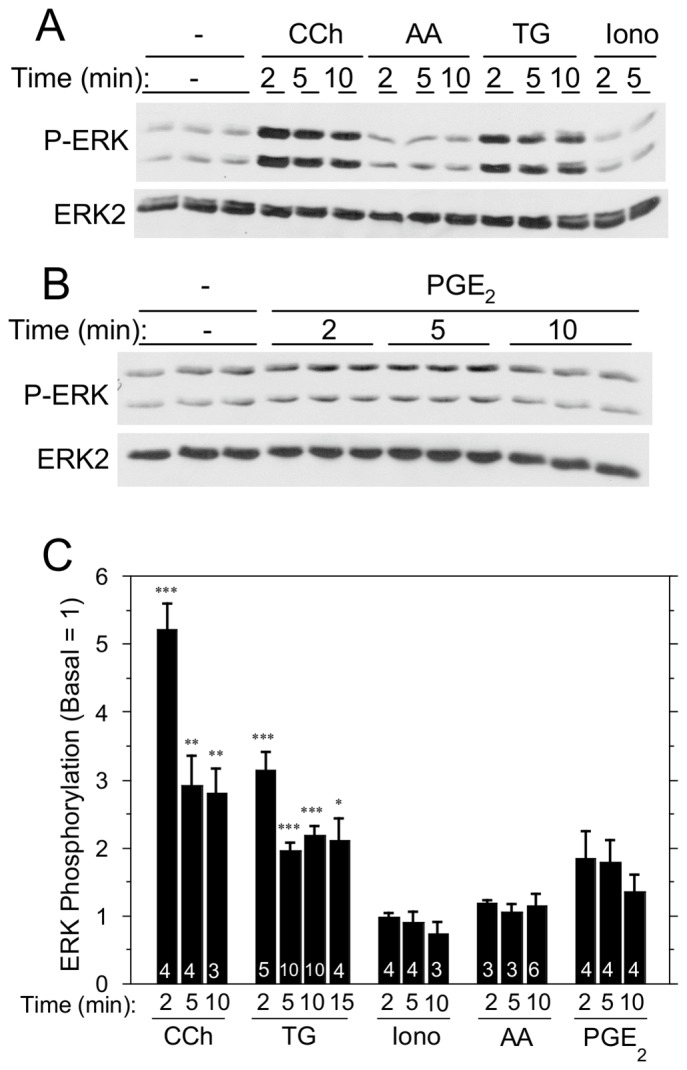
Time course of the effects of [Ca^2+^]_i_-elevating and other agents on ERK1/2 phosphorylation in rat parotid acinar cells. A. Comparison of effects of carbachol (10 µM), arachidonic acid (8 µM), TG (1 µM), and ionomycin (1 µM). B. Effect of PGE_2_ (10 µM). C. Quantitative comparison of multiple agents on ERK1/2 phosphorylation relative to basal. Number of individual experiments is indicated at bottom of the bars. ***p<0.001, **p<0.01, *p<0.05 compared to basal.

SOCE plays a critical role in promoting fluid secretion and saliva formation in salivary gland cells [10] Since changes in ion transport protein activation [32], [33], [34] and changes in [Ca^2+^]_i_ can initiate or modulate cell signaling events in a variety of cells, we compared the effects of [Ca^2+^]_i_ elevation by SOCE and other mechanisms on ERK1/2 signaling in rat parotid acinar cells. TG, which inhibits Ca^2+^ uptake into the endoplasmic reticulum Ca^2+^ stores and thereby initiates SOCE, produced an increase in ERK1/2 activation (phosphorylation) within 2 min (Figure 1A,C). The muscarinic receptor ligand carbachol (CCh), which increases [Ca^2+^]_i_ in rat parotid acinar cells by activating G-protein-coupled M3 muscarinic receptors, increased ERK1/2 phosphorylation as rapidly as did TG. Of note, the elevation of [Ca^2+^]_i_ by exposure of cells to the Ca^2+^ ionophore ionomycin did not activate ERK1/2. The exposure of cells to arachidonic acid (AA), which increases Ca^2+^ entry into salivary gland and other cells via ARC channels [14], [27], did not activate ERK1/2. PGE_2_, a product of arachidonic acid metabolism, also did not produce significant changes in ERK1/2 phosphorylation (Figure 1B,C). These results indicate that increases in [Ca^2+^]_i_ *per se* do not activate ERK1/2 in these cells [30], [35], and suggest that Ca^2+^ entry via SOCE is unique in its activation of ERK1/2.

### TG and Ca^2+^ Ionophore both Activate ERK1/2 in Salivary Gland Epithelial Cell Lines

**Figure 2:**
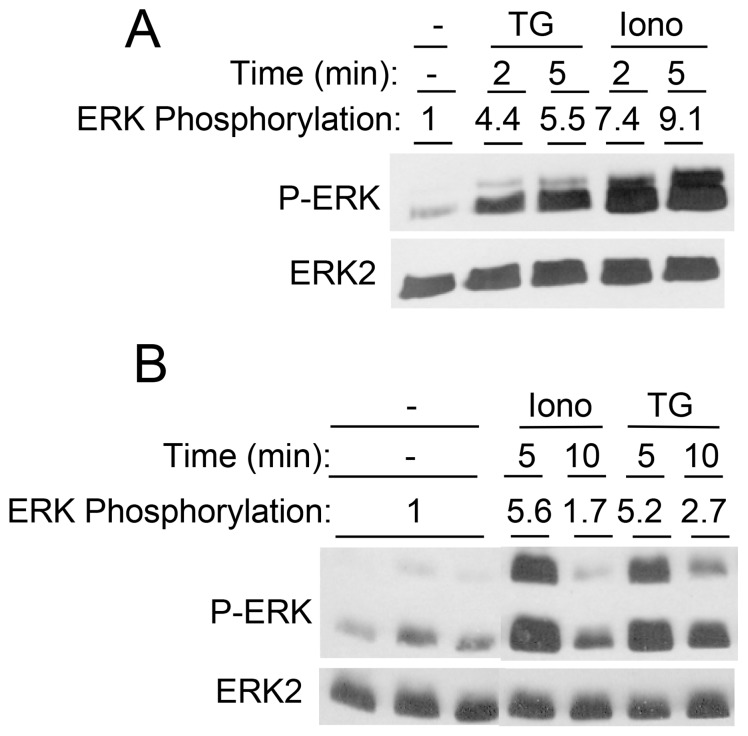
Time course of the effects of [Ca^2+^]_i_-elevating stimuli on ERK1/2 phosphorylation in salivary gland cell lines. HSY cells (A) and Par-C10 cells (B) were exposed to TG (1 µM) and ionomycin (1 µM) for various times, as indicated. Values shown are for ERK1/2 phosphorylations (normalized to total ERK2) relative to basal conditions.

We also examined the effects of TG on several salivary gland cell lines. TG increased ERK1/2 phosphorylation in Par-C10 cells, an immortalized rat parotid acinar cell line, and in HSY cells, a human parotid cancer cell line (Figure 2). However, ionomycin also increased ERK1/2 phosphorylation in both cell lines even though it did not do so in native rat parotid acinar cells (Figure 1). Thus, ERK1/2 was activated by global increases in [Ca^2+^]_i_ *per se* in these cell lines, responses that were different from those observed in native rat parotid acinar cells.

### Regulation of ERK1/2 by SOCE in Rat Parotid Acinar Cells

**Figure 3:**
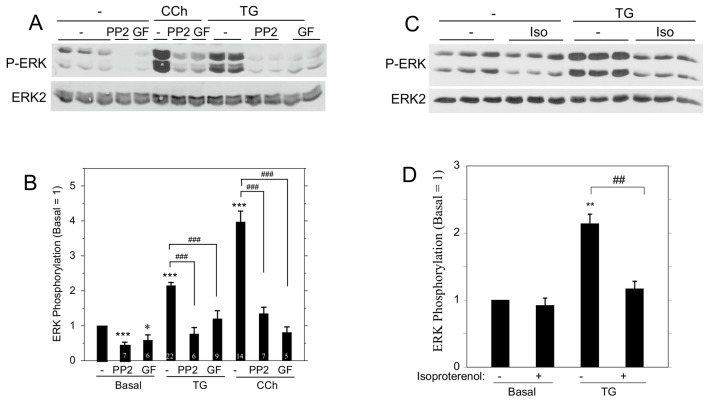
Modulation of ERK1/2 activation in rat parotid acinar cells by various signaling molecules. A. ERK1/2 phosphorylation was examined in cells that were pre-treated for 10 min with the SRC inhibitor PP2 (10 µM) or the PKC inhibitor GF109203X (10 µM), and then exposed to carbachol (10 µM, 2 min) or TG (1 µM, 10 min). B. Quantitative analysis of the effects of PP2 and GF109203X on ERK1/2 phosphorylation relative to basal conditions (vehicle). Number of individual experiments is indicated at bottom of the bars. *p<0.05, ***p<0.001 compared *to* basal; ###p<0.001 as indicated. C. ERK1/2 phosphorylation was examined in cells pre-treated with isoproterenol (0.1 µM) for 1 min prior to TG (1 µM, 2 min) or vehicle. D. Quantitative analysis of the ERK1/2 phosphorylation relative to basal (n = 3). **p<0.01 compared to basal; ##p<0.01 as indicated.

We compared the contributions of upstream kinases to ERK1/2 phosphorylation by TG and carbachol (Figure 3A,B). The SRC inhibitor PP2 and the PKC inhibitor GF109203X each blocked ERK1/2 phosphorylation promoted by TG and carbachol. These results indicate that SRC and PKC are both involved in the ERK1/2 activation pathway by these agents. We also found that the β-adrenergic receptor agonist isoproterenol blocked the TG-initiated ERK1/2 activation (Figure 3C,D), similar to our previous observations that isoproterenol blocks ERK1/2 phosphorylation downstream of the stimulation of various rat parotid acinar cell receptors, including the M3R, EGF receptor, and P2X_7_R [31]. These findings suggest that SOCE shares many similarities with receptors in its activation of cell signaling proteins.

### Dependence of ERK1/2 Activation on Extracellular Ca^2+^

**Figure 4:**
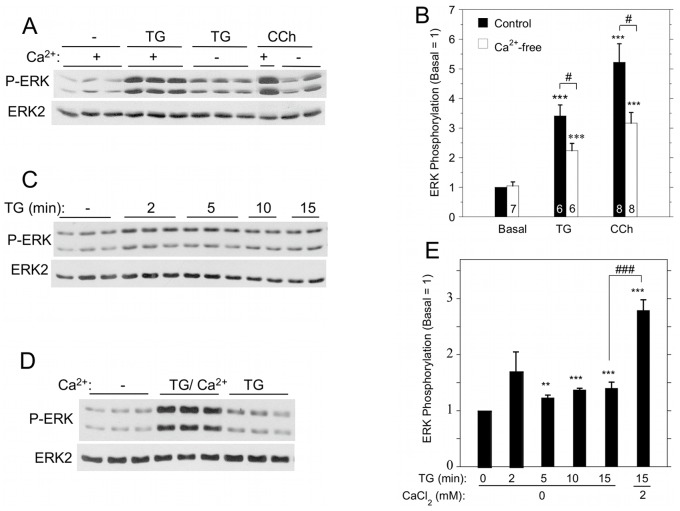
Contribution of extracellular Ca^2+^ to ERK1/2 activation by TG and carbachol in rat parotid acinar cells. A. Cells were suspended in the absence or presence (1.8 mM) of Ca^2+^, and exposed to TG (1 µM) or carbachol (10 µM) for 2 min. B. Quantitative analysis of ERK1/2 phosphorylation in conditions shown in Figure 4A. ***p<0.001 compared to basal, #p<0.05 as indicated. C. Time course of ERK1/2 phosphorylation in cells exposed to TG (1 µM) in the absence of Ca^2+^. D. Comparison of ERK1/2 phosphorylation in cells in Ca^2+^-free conditions and exposed to TG (1 µM, 15 min) or exposed to TG (1 µM, 15 min) followed by Ca^2+^ (1 mM, 2 min) to initiate SOCE. E. Quantitative analysis of ERK1/2 phosphorylation for conditions shown in Figure 4C and 4D. **p<0.01, ***p<0.001 compared to basal; ###p<0.001 as indicated. N = 3–16.

To determine whether TG-initiated ERK1/2 activation was dependent on Ca^2+^ entry, we performed experiments in the absence of extracellular Ca^2+^ to block increases in [Ca^2+^]_i_ via SOCE. TG promoted small but significant increases in ERK1/2 phosphorylation in cells suspended in the absence of extracellular Ca^2+^, but TG produced larger increases in the presence of Ca^2+^ (Figure 4A,B). The activation of ERK1/2 by carbachol also displayed a dependence on extracellular Ca^2+^ (Figure 4A, B). We examined this more closely and found that in the absence of Ca^2+^, TG produced a larger increase in ERK1/2 phosphorylation after 2 min than at later times up to 15 min of exposure (Figure 4C,E). Notably, there was a large SOCE-dependent increase in ERK1/2 phosphorylation upon the addition of Ca^2+^ for 2 min to cells previously exposed to TG for 15 min in Ca^2+^-free solution (Figure 4D,E).

**Figure 5:**
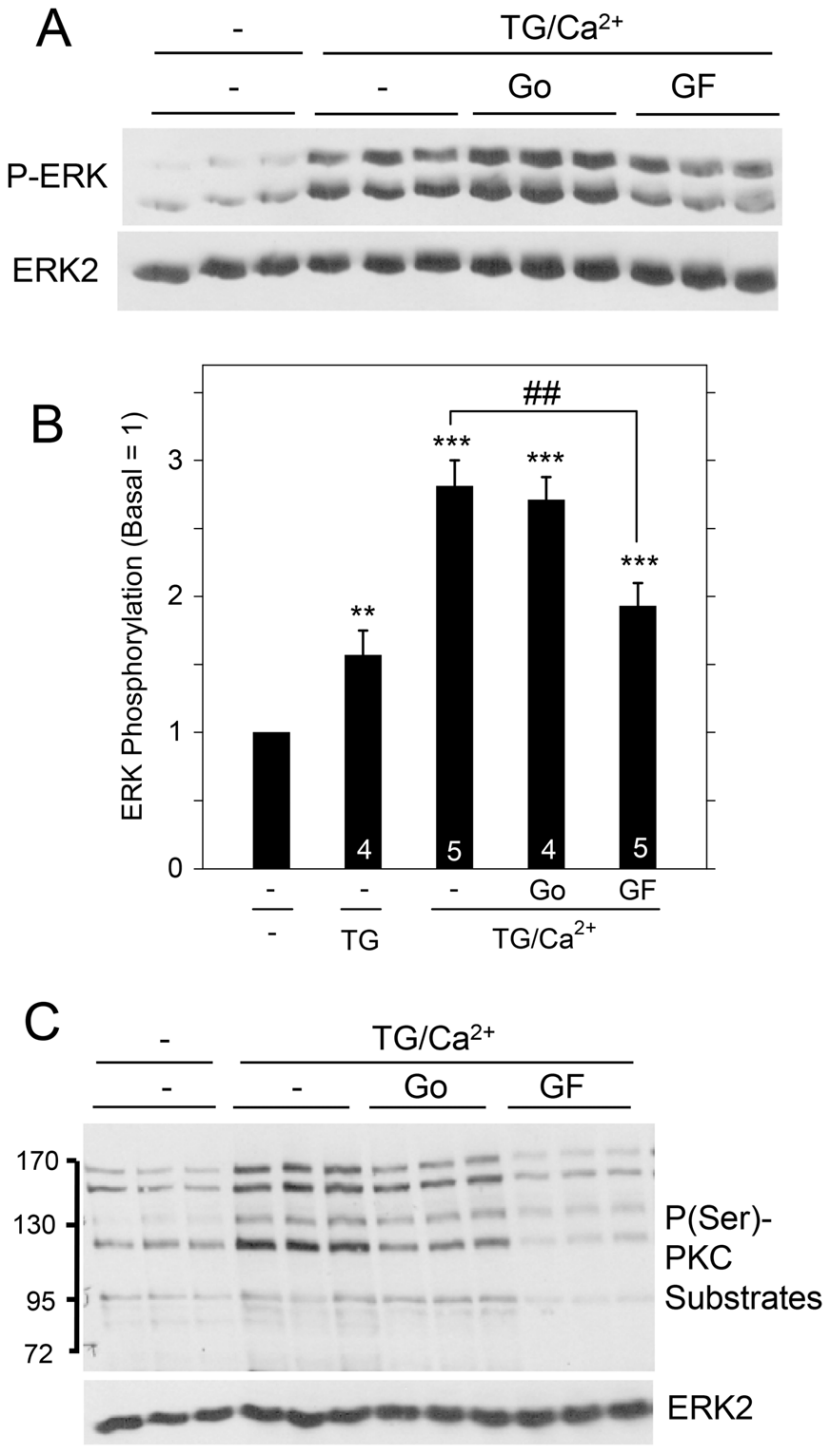
Contribution of PKC proteins to ERK1/2 activation by SOCE in rat parotid acinar cells. Cells were suspended in the absence of extracellular Ca^2+^, exposed to TG (1 µM) for 15 min in the absence or presence of GF109203X (10 µM) and Go6976 (1 µM), and then exposed to CaC1_2_ (1 mM, 2 min). A. Effect of PKC inhibitors on ERK1/2 phosphorylation due to Ca^2+^ entry into cells. B. Quantitative analysis of ERK1/2 phosphorylation for conditions shown in Figure 5A and TG (1 µM, 15 min) alone. **p<0.01, ***p<0.001 compared to basal; ##p<0.01 as indicated. C. Effect of PKC inhibitors in blocking the phosphorylation of PKC substrates in TG-treated cells. Conditions are identical to those shown in Figure 5A. ERK2 was a loading control.

Using this protocol (*i.e*., exposing cells to Ca^2+^ for 2 min after TG for 15 min TG in Ca^2+^-free solution), we reexamined the contribution of PKC to ERK1/2 activation (Figure 5). Go6976, which inhibits cPKC family members (PKCα, PKCβ, PKCγ), was ineffective in blocking the increase in ERK1/2 phosphorylation by SOCE; in contrast, GF109203X, which inhibits cPKC and nPKC (PKCδ, PKCε, PKCη, PKCθ) family members, significantly blocked ERK1/2 activation. GF109203X was also more effective than Go6976 in blocking the phosphorylation of PKC substrates downstream of PKC activation by TG. These results suggest that nPKC proteins are involved in the activation of ERK1/2 by TG, similar to their involvement in ERK1/2 activation by carbachol [30].

### Agents that Block SOCE also Reduce the Activation of ERK1/2

**Figure 6:**
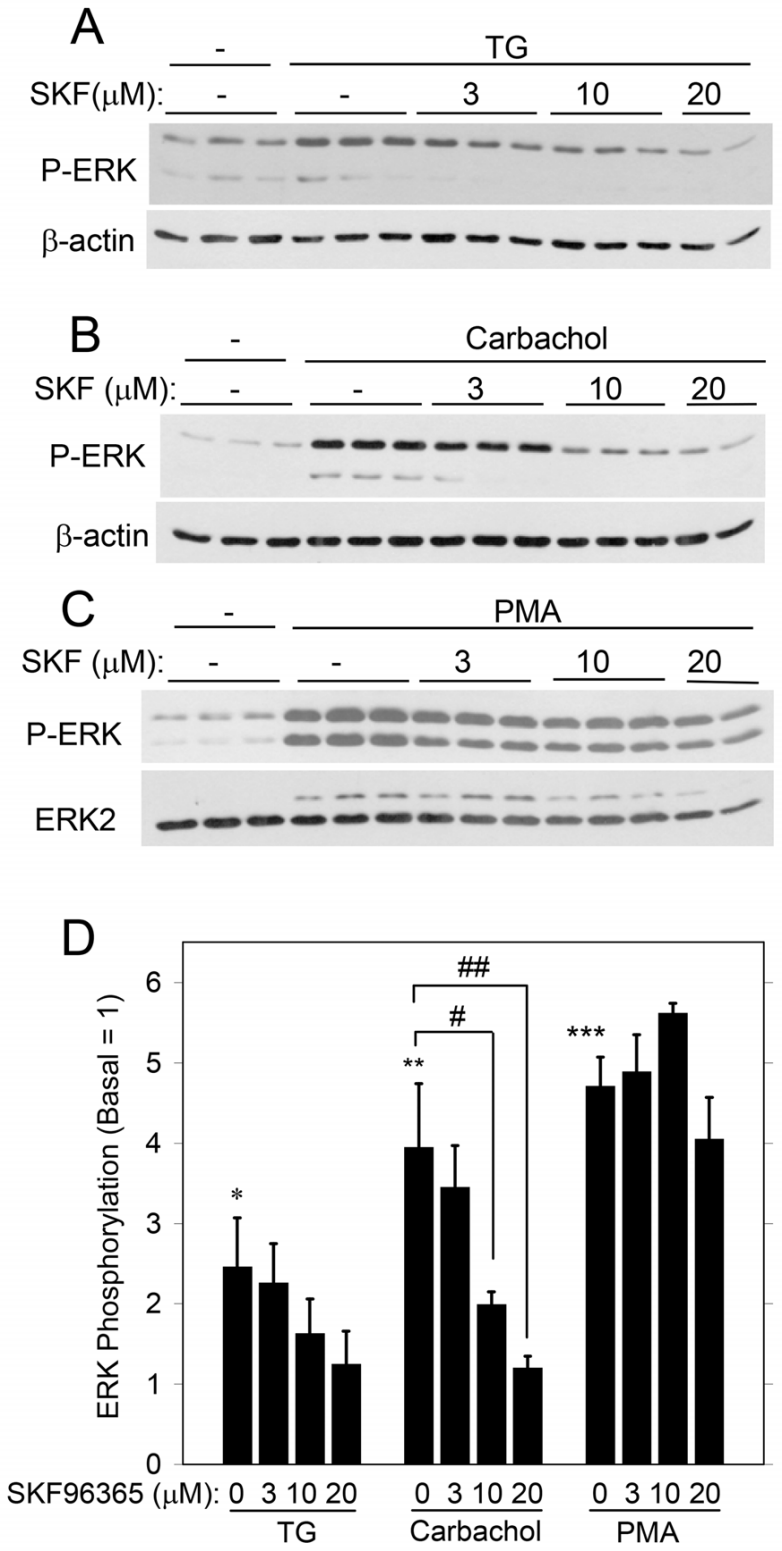
Effect of the SOCE blocker SKF96365 on ERK1/2 activation in rat parotid acinar cells. Cells were exposed to different concentrations of SKF96365 (3–20 µM) for 10 min, and subsequently treated for 2 min with the following: A, TG (1 µM); B, carbachol (10 µM), and C, PMA (100 nM). D. Quantitative analysis of ERK1/2 phosphorylation for conditions shown in Figure 6A,B,C. *p<0.05, **p<0.01, ***p<0.001 compared to basal. #p<0.05, ##p<0.01 as indicated. N = 3–5.

We examined the effects of several blockers of SOCE to evaluate further the contribution of SOCE to ERK1/2 activation. SKF96365 [36] reduced the activation of ERK1/2 by carbachol in a concentration-dependent manner (Figure 6), and there was a trend that it also reduced the activation by TG. (SKF96365 (20 µM) produced a significant reduction using Student’s t-test, but not when the data were analyzed using ANOVA). Notably, SKF96355 was not effective in blocking the activation of ERK1/2 by the phorbol ester PMA, which directly activates PKC. Since TG and carbachol, but not PMA, promote SOCE, the inhibitory effect of SKF96365 on ERK1/2 is consistent with the involvement of SOCE. SKF96365 blocks TRPC proteins [37], which along with other proteins (Orai1, STIM1) make up the complex that conducts SOCE in salivary gland cells [1], [23].

**Figure 7:**
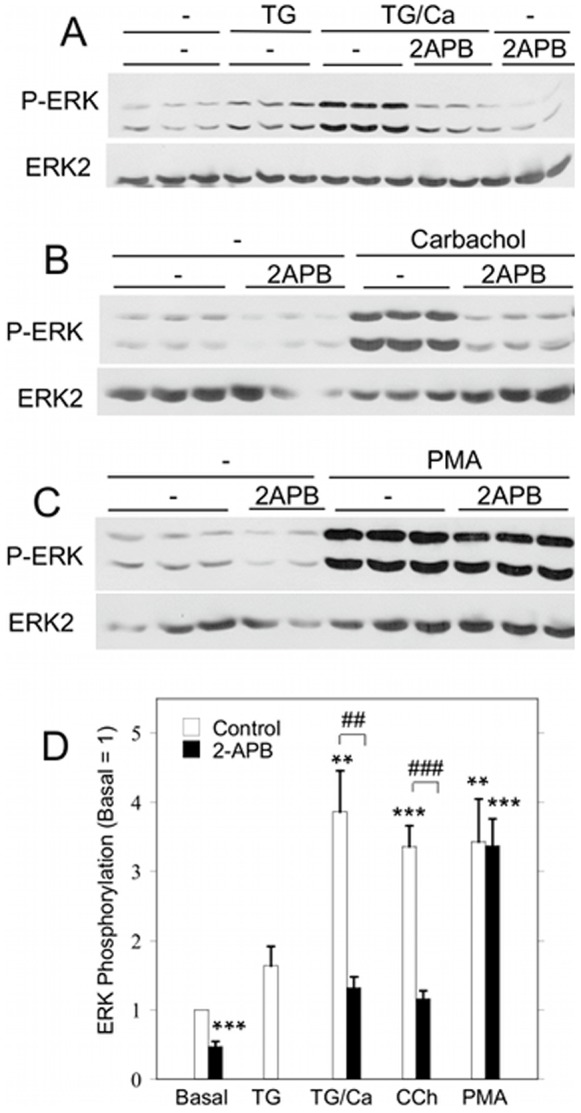
Effect of the SOCE blocker 2-APB on ERK1/2 phosphorylation in rat parotid acinar cells. A. Cells were suspended in Ca^2+^-free solution, and TG (1 µM) alone was added for 15 min (TG) or 1 mM CaC1_2_ was added for 2 min after 15 min of TG (TG/Ca^2+^). B, C. Cells suspended in 1.8 mM CaCl_2_ were treated with carbachol (10 µM, 2 min) or PMA (100 nM, 2 min) in the absence or presence of 2-APB. Cells were exposed to 2-APB (20 µM) or vehicle (control) for 5 min prior to treatment with stimulating agents. D. Quantitative analysis of ERK1/2 phosphorylation for the conditions shown in Figures 7A,B,C relative to basal control conditions. **p<0.01, ***p<0.001 compared to basal. ##p<0.01, ###p<0.001 as indicated. N = 5–8.

**Figure 8:**
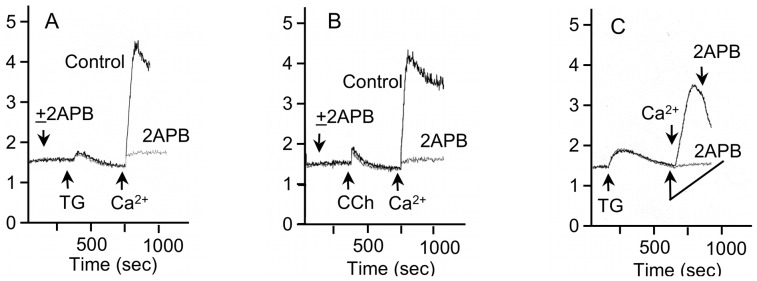
Inhibition of SOCE by 2-APB in rat parotid acinar cells. A, B. 2-APB (20 µM) blocked the entry of Ca^2+^ into Fura2-loaded cells in which Ca^2+^ stores were depleted by the addition of TG (1 µM) (Figure 8A) or carbachol (10 µM) (Figure 8B) to cells in Ca^2+^-free solution. Vehicle was added to the control cells. C. 2-APB blocks Ca^2+^ entry after depletion of Ca^2+^ stores by TG (1 µM) and after the increase in [Ca^2+^]_i_ produced by addition of 1 mM CaCl_2_ to store-depleted cells. After depletion of Ca^2+^ stores and the return of [Ca^2+^]_i_ to baseline levels, 2-APB (20 µM) was added (gray line) 1 min prior to the addition of 1 mM CaCl_2_. After the peak increase in Ca^2+^ produced by addition of 1 mM CaCl_2_ after store-depletion under control conditions (black line), 2-APB produced an immediate decrease in the level of [Ca^2+^]_i_. The ordinate axis is 340/380-nm Fura-2 fluorescence excitation ratio. Shown are individual traces of one experiment, which are representative of at least 3 experiments.

2-APB, which blocks SOCE by blocking the complex formation of STIM1, Orai1, and other proteins [24], [38], [39], inhibited ERK1/2 phosphorylation initiated by TG and carbachol, but not PMA (Figure 7). This is consistent with a role for SOCE in ERK1/2 activation by TG and carbachol. Since 2-APB may also block the IP_3_R [40], this could lead to a reduction in SOCE via an indirect mechanism: a decrease in the depletion of the endoplasmic reticulum Ca^2+^ stores. However, 2-APB did not block the TG-initiated (Figure 8A) or carbachol-initiated (Figure 8B) Ca^2+^ release from intracellular Ca^2+^ stores, but it did block the subsequent entry of Ca^2+^ when extracellular Ca^2+^ was added to store-depleted cells in Ca^2+^-free solution. 2-APB also blocked SOCE when it was added after the depletion of Ca^2+^ stores by TG, and it produced an immediate decrease in [Ca^2+^]_i_ during the peak elevation of [Ca^2+^]_i_ due to SOCE (Figure 8C), as demonstrated in other studies [41]. These data are consistent with reports that the main inhibitory effect of 2-APB is on SOCE rather than on the IP_3_R [40].

**Figure 9:**
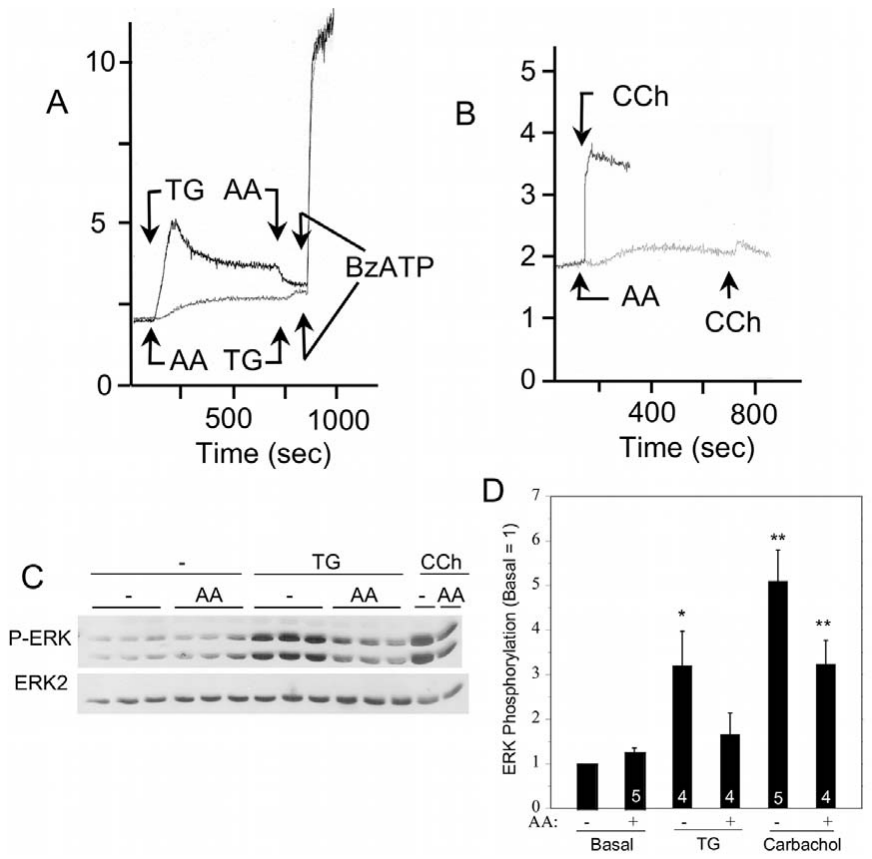
Effect of arachidonic acid (AA) on SOCE and ERK1/2 activation in rat parotid acinar cells. A. After TG (1 µM) initiates SOCE and the elevation of [Ca^2+^]_i_ (black line), the sustained [Ca^2+^]_i_ level is reduced by AA (8 µM); however, AA does not block the entry of Ca^2+^ via P2X_7_R activation by BzATP (10 µM). Similarly, ARC channel activation by AA (8 µM) promotes Ca^2+^ entry and an increase in [Ca^2+^]_i_ levels to a steady plateau (gray line), and this blocks the large increase in [Ca^2+^]_i_ by TG (1 µM) but not by BzATP (10 µM). B. ARC channel activation by AA (8 µM) also blocks CCh (10 µM)-initiated SOCE. In A and B individual traces of Fura2-loaded cells from one experiment are representative of at least 3 experiments. The ordinate axis is 340/380-nm Fura-2 fluorescence excitation ratio. C. Activation of ARC by AA (8 µM, 10 min) had no effect on ERK1/2 phosphorylation but affected the increases produced by TG (1 µM, 2 min) and carbachol (10 µM, 2 min). D. Quantitative analysis of ERK1/2 phosphorylation for the conditions shown in Figure 9C relative to basal control conditions. *p<0.05, **P<0.01 compared to basal. Number of individual experiments is indicated at bottom of the bars.

Rat parotid acinar cells exposed to arachidonic acid exhibit a slow increase in [Ca^2+^]_i_ due to Ca^2+^ entry via ARC channels, and arachidonic acid blocked the TG- and carbachol-initiated increases in [Ca^2+^]_i_ via SOCE (Figure 9A,B). If added during the SOCE-mediated sustained elevation of [Ca^2+^]_i_ by TG, arachidonic acid reduced the level of [Ca^2+^]_i_. In contrast, arachidonic acid did not block the increase in [Ca^2+^]_i_ due to the entry of Ca^2+^ via the P2X_7_R, which is a nonselective cation channel in rat parotid acinar and other cells [9], [42]. To examine the contribution of SOCE to ERK1/2 activation without resorting to chemical blockers, we used arachidonic acid. The exposure to arachidonic acid alone did not affect ERK1/2 phosphorylation (Figure 9C,D; see also Figure 1), and there was a trend that arachidonic acid reduced ERK1/2 activation by the subsequent addition of TG and carbachol. (When this data was subjected to Student’s *t*-test instead of ANOVA, ARC channel activation produced a statistically significant reduction of SOCE-initiated ERK1/2 phosphorylation by TG and carbachol).

## Discussion

These studies were conducted to examine the selectivity of cell signaling downstream of various mechanisms that increase [Ca^2+^]_i_ in rat parotid acinar cells. The main physiological function of these salivary gland cells is to initiate fluid secretion and saliva formation in response to neurotransmitters that bind to and activate phospholipase C-linked G-protein-coupled receptors. This results in the generation of the second messenger IP_3_ and the promotion of Ca^2+^ release from intracellular stores and the subsequent Ca^2+^ entry across the plasma membrane via SOCE. The elevation of [Ca^2+^]_i_ opens Ca^2+^-sensitive K^+^ and Cl^−^ channels that contribute to net fluid secretion into the acinar lumen [2], [28], [43]. Ca^2+^-sensitive ion channels and subsequent ionic changes and secondary ionic movements via other ion transport proteins can be initiated by [Ca^2+^]_i_ elevation via multiple pathways, including increases initiated by G-protein-coupled receptors, P2X_7_Rs/ion channels, and Ca^2+^ ionophores [2], [7], [10], [11], [12].

The data demonstrate that ERK1/2 phosphorylation is increased by the TG-initiated elevation of [Ca^2+^]_i_ by SOCE in the absence of G-protein-coupled receptor activation. In the absence of extracellular Ca^2+^, the increase in [Ca^2+^]_i_ is modest and also is transient. In contrast, a large increase in ERK1/2 phosphorylation upon the addition of extracellular Ca^2+^ to store-depleted cells accompanies the large increase in [Ca^2+^]_i_ via SOCE under these conditions.

TG-promoted SOCE also increased ERK1/2 activation in human platelets [44], [45] and vascular smooth muscle cells [46]; and TG-initiated ERK1/2 activation in B cells was dependent on extracellular Ca^2+^ [47]. However, not all effects of TG on ERK1/2 phosphorylation are necessarily due to its effects on SOCE; some are due to its effects on endoplasmic reticulum stress [48], [49].

Among the multiple mechanisms of Ca^2+^ elevation by Ca^2+^ entry that we investigated, SOCE was unique in its stimulation of ERK1/2 activation in rat parotid acinar cells. Ca^2+^ ionophores and ARC channel activation did not increase ERK1/2 phosphorylation, demonstrating that that Ca^2+^ *per se* did not activate the cell signaling proteins, a finding that is much different from the central role of Ca^2+^ in stimulating net ion movements and ion transporters in these and other salivary gland cells. The regulation of ERK1/2 activation by SOCE in rat parotid acinar cells was very similar to that promoted by the activation of the muscarinic and other receptors, including the P2X_7_R [35]. The increases in ERK1/2 phosphorylation by SOCE were dependent on PKC activity, similar to what we previously demonstrated for the activation of the M3 muscarinic receptor and P2X_7_R/channel [30], [35]. The TG-initiated ERK1/2 phosphorylation was also blocked by exposure of the cells to the β-adrenergic agonist isoproterenol. This treatment likely blocks the ERK1/2 signaling cascade at the level of Raf, accounting for its inhibition of ERK1/2 phosphorylation downstream of other receptors in rat parotid acinar cells [31].

In rat parotid acinar cells, ERK1/2 is activated by diacylglycerol production, and thus the phorbol ester PMA (a diacylglycerol mimetic) produces large increases in ERK1/2 phosphorylation [30]. We do not know if SOCE stimulates ERK1/2 via phospholipase C-mediated diacylglycerol production. However, among multiple Ca^2+^ entry pathways, SOCE may uniquely activate phospholipase C in a manner analogous to its activation of AC8, which is not reproduced by ARC channel activation or Ca^2+^ ionophores [13], [14], [50]. AC8 and SOCE channels have a close spacial relationship [50]. Since ionomycin did not activate ERK1/2 in native rat parotid acinar cells, this suggests that global increases in Ca^2+^ did not activate phospholipase C and produce diacylglycerol (and IP_3_) from PIP_2_ hydrolysis, as it can do in some other systems [51]. Notably, ionomycin did initiate ERK1/2 activation in human and rat salivary gland cells lines (Figure 2), demonstrating that salivary gland cell lines and native salivary gland cells do not always respond in a similar manner, as we’ve noted when we analyzed other biological events in previous studies [31], [33].

2-APB and SKF96365 inhibit SOCE in salivary gland cells [1], [52], [53], [54] and blocked increases in ERK1/2 phosphorylation by TG and carbachol to a varying degree. Of note, ARC channels are not sensitive to 2-APB [55]. Although these compounds have some limitations in their specificity [40], they did not block the stimulatory effect of PMA (Figure 6, 7), which was used as a control to check for off-target effects. In a related study, the TG-initiated phosphorylation of ERK1/2 and its downstream effector Ca^2+^/cAMP response element binding protein (CREB) was blocked by 2-APB in vascular smooth muscle cells, consistent with ERK1/2 and CREB activation by SOCE [46]. Several studies reported that Ca^2+^ entry via ARC channels and SOCE are reciprocally regulated. Shuttleworth and collaborators [5] observed that Ca^2+^ entry via ARC occurs at low receptor agonist concentration, and that higher concentrations of agonist activate SOCE and produce high sustained levels of cytosolic Ca^2+^ that block ARC. Putney and collaborators [56] reported that activation of either SOCE or ARC blocked the subsequent activation of the alternate Ca^2+^ entry pathway, which is consistent with our observations of the negative effect of ARC channels on changes in [Ca^2+^]_i_ via SOCE.

ERK1/2 activation by SOCE could have several downstream effects, including initiating a positive feedback on SOCE itself. ERK1/2 had a positive effect on SOCE in various tissues [44], [45], [57], as indicated by the reduction of SOCE in cells treated with MEK inhibitors that blocked ERK1/2 phosphorylation. The positive effects of ERK1/2 on SOCE may involve STIM1 phosphorylation, since STIM1 phosphorylation was detected using a mass spectrometry approach, ERK1/2 phosphorylated STIM1 *in vitro*, and a triple mutation of serine to alanine mutation in all three ERK1/2 target sites on STIM1 (S575, S608, S621) resulted in a reduction in SOCE [58]. In contrast, the phosphorylation of STIM1 on other several serine residues during mitosis, perhaps involving cyclin dependent kinase 1 and other kinases, resulted in a decrease in SOCE [59]. The phosphorylation of STIM1 on tyrosine residues increased the entry of Ca^2+^ via SOCE [60]. Orai also has been reported to be the substrate of kinases such as PKC [61], which produces a decrease in SOCE activity. In preliminary experiments we did not find any evidence that ERK1/2 affected SOCE in rat parotid acinar cells: the entry of Ca^2+^ into cells in which Ca^2+^ stores were first depleted by TG (as in Figure 8) was not affected when ERK1/2 activation was blocked by exposing cells to MEK inhibitors to block ERK1/2 phosphorylation (not shown).

TG and other agents that cause endoplasmic reticulum stress produced oscillations of ERK1/2 and c-Jun N-terminal kinase 1/2 (JNK), and these oscillations were probably SOCE-independent [49]. In contrast to our findings and those cited above, the TG-promoted release of Ca^2+^ from endoplasmic reticulum stores activated ERK1/2 in a SOCE-independent manner in endothelial cells, and the activated ERK1/2 phosphorylated and activated endothelial nitric oxide synthase [62]. ERK1/2 was reported to reduce Ca^2+^ entry by phosphorylating the IP_3_ type 1 receptor, which inhibited the release of Ca^2+^ stores and thus, also, the subsequent SOCE [63]. In addition, when TG was used as an inducer of endoplasmic reticulum stress, it increased the phosphorylation of the stress proteins p38 MAPK and altered the expression of various genes on a longer time scale (hours) compared to that (minutes) that was monitored in the present studies [64].

## Conclusions

In summary, we observed that ERK1/2 was activated in rat parotid acinar cells by Ca^2+^ entry via receptor-independent SOCE, but not by Ca^2+^ entry via ARC channels and Ca^2+^ ionophore. TG and carbachol shared multiple characteristics as agents that increased ERK1/2 phosphorylation, including the kinetics and significant increases above basal levels, the dependences on similar signaling and regulatory proteins, and the reductions by agents and conditions that block SOCE. The data suggest that part of the receptor-initiated stimulation of ERK1/2 is dependent on SOCE; in addition, we suggest that the receptor-independent stimulation of ERK1/2 may rely on phospholipase C activation. Calcium ionophore and the activation of ARC channels were insufficient to activate ERK1/2; thus our findings indicate that the activation of cell signaling proteins by Ca^2+^ entry into native rat cells is much more discriminatory than the activation of ion transport proteins by [Ca^2+^]_i_. These studies demonstrate the selective activation of cellular responses downstream of a specific mechanisms of Ca^2+^ entry into these cells, and how SOCE may contribute to cellular processes beyond to its role in elevating [Ca^2+^]_i_.
